# Six-lead electrocardiography compared to single-lead electrocardiography and photoplethysmography of a wrist-worn device for atrial fibrillation detection controlled by premature atrial or ventricular contractions: six is smarter than one

**DOI:** 10.3389/fcvm.2023.1160242

**Published:** 2023-06-09

**Authors:** Justinas Bacevicius, Neringa Taparauskaite, Ricardas Kundelis, Daivaras Sokas, Monika Butkuviene, Guoste Stankeviciute, Zygimantas Abramikas, Aiste Pilkiene, Ernestas Dvinelis, Justina Staigyte, Julija Marinskiene, Deimile Audzijoniene, Marija Petrylaite, Edvardas Jukna, Albinas Karuzas, Vytautas Juknevicius, Rusne Jakaite, Viktorija Basyte-Bacevice, Neringa Bileisiene, Ignas Badaras, Margarita Kiseliute, Gintare Zarembaite, Modestas Gudauskas, Eugenijus Jasiunas, Linda Johnson, Vaidotas Marozas, Audrius Aidietis

**Affiliations:** ^1^ Institute of Clinical Medicine, Faculty of Medicine, Vilnius University, Vilnius, Lithuania; ^2^ Center of Cardiology and Angiology, Vilnius University Hospital Santaros Klinikos, Vilnius, Lithuania; ^3^ Biomedical Engineering Institute, Kaunas University of Technology, Kaunas, Lithuania; ^4^ Center of Informatics and Development, Vilnius University Hospital Santaros Klinikos, Vilnius, Lithuania; ^5^ Department of Clinical Sciences, Malmö, Lund University, Lund, Sweden; ^6^ Electronics Engineering Department, Kaunas University of Technology, Kaunas, Lithuania

**Keywords:** wearable, smartWatch, multiple-lead ECG, telemedicine, mHealth, remote monitoring, digital

## Abstract

**Background:**

Smartwatches are commonly capable to record a lead-I-like electrocardiogram (ECG) and perform a photoplethysmography (PPG)-based atrial fibrillation (AF) detection. Wearable technologies repeatedly face the challenge of frequent premature beats, particularly in target populations for screening of AF.

**Objective:**

To investigate the potential diagnostic benefit of six-lead ECG compared to single-lead ECG and PPG-based algorithm for AF detection of the wrist-worn device.

**Methods and results:**

From the database of DoubleCheck-AF 249 adults were enrolled in AF group (*n* = 121) or control group of SR with frequent premature ventricular (PVCs) or atrial (PACs) contractions (*n* = 128). Cardiac rhythm was monitored using a wrist-worn device capable of recording continuous PPG and simultaneous intermittent six-lead standard-limb-like ECG. To display a single-lead ECG, the six-lead ECGs were trimmed to lead-I-like ECGs. Two diagnosis-blinded cardiologists evaluated reference, six-lead and single-lead ECGs as “AF”, “SR”, or “Cannot be concluded”. AF detection based on six-lead ECG, single-lead ECG, and PPG yielded a sensitivity of 99.2%, 95.7%, and 94.2%, respectively. The higher number of premature beats per minute was associated with false positive outcomes of single-lead ECG (18.80 vs. 5.40 beats/min, *P *< 0.01), six-lead ECG (64.3 vs. 5.8 beats/min, *P *= 0.018), and PPG-based detector (13.20 vs. 5.60 beats/min, *P *= 0.05). Single-lead ECG required 3.4 times fewer extrasystoles than six-lead ECG to result in a false positive outcome. In a control subgroup of PACs, the specificity of six-lead ECG, single-lead ECG, and PPG dropped to 95%, 83.8%, and 90%, respectively. The diagnostic value of single-lead ECG (AUC 0.898) was inferior to six-lead ECG (AUC 0.971) and PPG-based detector (AUC 0.921). In a control subgroup of PVCs, the specificity of six-lead ECG, single-lead ECG, and PPG was 100%, 96.4%, and 96.6%, respectively. The diagnostic value of single-lead ECG (AUC 0.961) was inferior to six-lead ECG (AUC 0.996) and non-inferior to PPG-based detector (AUC 0.954).

**Conclusions:**

A six-lead wearable-recorded ECG demonstrated the superior diagnostic value of AF detection compared to a single-lead ECG and PPG-based AF detection. The risk of type I error due to the widespread use of smartwatch-enabled single-lead ECGs in populations with frequent premature beats is significant.

## Introduction

Atrial fibrillation (AF) is an arrhythmia that can lead to various cardiovascular events including ischemic stroke and heart failure, especially if undiagnosed or not treated adequately (1). AF is the most common arrhythmia in the world with the latest approximate prevalence of 60 million patients and contributes to >8 million disability-adjusted life years (2). While the prevalence of this disease increases, there is still a high percentage of undiagnosed cases (3, 4). This includes asymptomatic patients and patients who experience symptoms but the diagnosis of AF is not confirmed with a standard 12-lead electrocardiogram (ECG). As undiagnosed AF may pose potential risks to the patient and, in case of adverse cardiovascular events, additional burden to the health care system (5), early AF diagnosis and management is of crucial importance (6). To reduce the number of undiagnosed AF cases, systematic screening for AF should be considered in individuals aged ≥75 years (7). In addition, the new practical guide of the European Heart Rhythm Association (EHRA) upgraded the consensus statement to “may be beneficial” in individuals aged ≥65 years with comorbidities increasing the risk of stroke (as systematic screening by intermittent ECG) and in patients aged ≥65 years without comorbidities or <65 years with comorbidities (as opportunistic screening) (8).

Increasing numbers of wearable technologies facilitate the detection of AF in asymptomatic or undiagnosed symptomatic individuals and establish a clear hierarchy of diagnostic methods for AF screening. As a rule of thumb, photoplethysmography-based (PPG) devices are preferred to pulse palpation. However, if PPG screening is indicative of AF, only an ECG-based method should be used to confirm the diagnosis of AF and is preferred over PPG-based devices (8).

The key factor for high diagnostical accuracy for AF detection using a wearable device is the sufficient quality of ECG. When artifacts are present, conventional multiple-lead-ECG Holter monitoring demonstrates additional vectors of electrical activity and subsequently increases the chances of correct interpretation (9). However, the situation is different in a real-life setting, i.e., artifacts, noise, and the presence of other concomitant arrhythmias with irregular heart contractions, such as premature beats, are the most common challenges for AF detection in wearable-recorded ECGs (10).

Most current smartwatches share a common feature of recording a lead-I-like ECG. Our scientific group has introduced the first wrist-worn device, which combines a PPG-based algorithm for AF screening and intermittent 6-lead ECG recorded with no wires for AF confirmation (11). Whether multi-lead ECG recorded using a wrist-worn device brings an additional benefit for AF detection compared to single-lead ECG is unknown. The aim of this study is to compare the performance of single-lead and six-lead ECGs obtained using the wrist-worn device as well as the automatic PPG-based AF detector.

## Materials and methods

### Study design

This was a single-center, non-randomized substudy of DoubleCheck-AF with a prospective case-control model. A regional bioethics committee approved it with registration No. 158200-18/7-1052-557. The study is registered at ClinicalTrials.gov (NCT04281927).

Patients were recruited from both inpatient and outpatient wards of Cardiology Department at Vilnius University Hospital Santaros Klinikos at any time of the day. All the participants gave written informed consent before enrolment. Adult patients (18 to 99 years) diagnosed with AF or sinus rhythm (SR) with frequent PVCs or PACs were included in the study. Patients in SR with frequent PVCs and PACs were selected as a control group. Individuals with at least one ectopic beat in 2 min were classified as SR with frequent PVCs or PACs. Patients who did not give informed consent, had paced ventricular beats, other arrhythmias or stable SR were excluded from the study.

A sample of 435 patients was collected in the original DoubleCheck-AF study. For analysis of 2 × 2 contingency tables [degree of freedom (df) = 1], medium effect size (*w* = 0.3), *α* error probability = 0.05, and power (1 − *β*) = 0.95, we needed a sample size of 145 patients. In the current substudy, after the exclusion of the control subgroup of stable SR, the remaining subjects (*n* = 249) were sufficient to match the required sample size.

### Measurements

Cardiac rhythm was monitored using a wrist-worn device, detailly described by Bacevicius et al. (11), which provides continuous PPG-based AF monitoring and an intermittent, on-demand, six-lead ECG. Synchronously, reference ECG was registered using a validated Holter monitor (Bittium Faros, Bittium, Finland).

The PPG signals are analyzed using an embedded AF detector (12), which structure is inspired by the rhythm-based detector used for ECG signals (13). The algorithm relies on the analysis of peak-to-peak intervals using 8-beat long sliding window and includes blocks of signal quality assessment, peak-to-peak interval characterization, and suppression of non-AF rhythms such as ectopic beats, bigeminy, and respiratory sinus arrhythmia.

The main specifications of the wearable device are as follows. The sampling rate of the PPG signal is 100 Hz, the amplitude resolution is 18 bits, and the bandwidth is 0–50 Hz. The device can record green, red, and infrared light channels, although only the green channel was used in this application.

**Figure 1:**
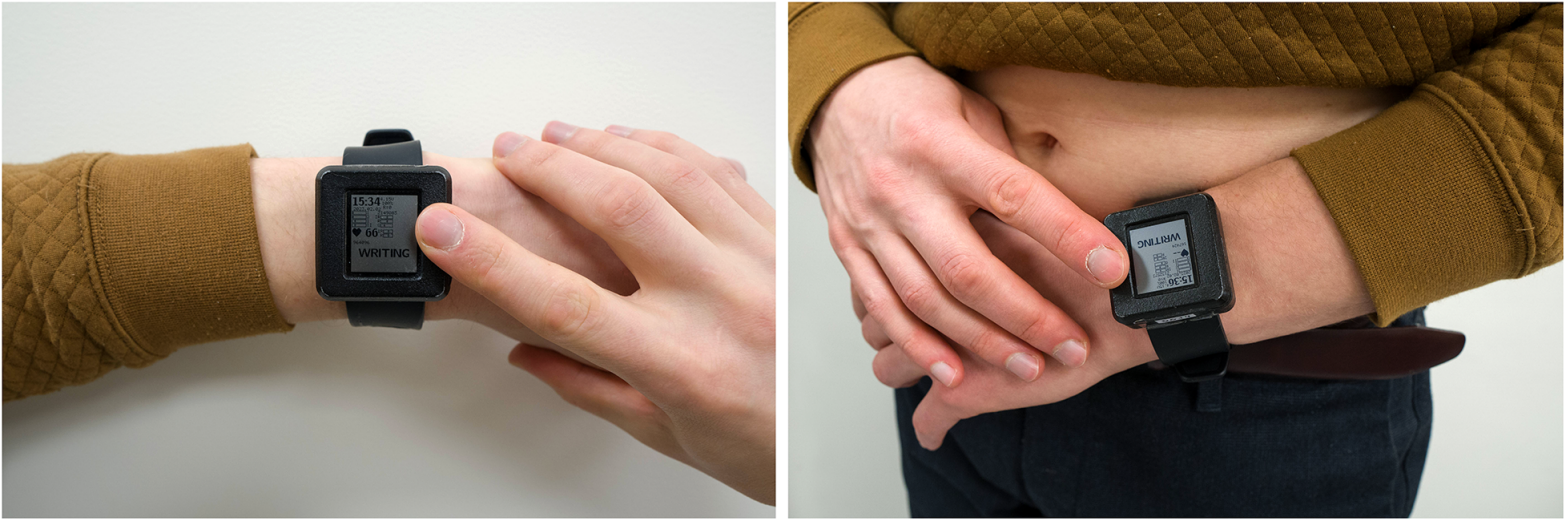
Acquiring of single-lead ECG (left panel) and six-lead ECG without any wires (right panel) with the use of the prototype device. The configuration of electrodes is displayed elsewhere (11).

The recorded ECG leads are similar to standard Einthoven-like limb leads, as they are measured by contact of three electrodes to the skin: two electrodes are on the outer surface (one electrode on top of the device enclosure, another electrode on the bracelet), and the third electrode is placed on the inner surface next to the PPG sensor (Figure 1). Additional three ECG leads (Goldberger augmented limb leads aVR, aVL, and aVF) were calculated from Einthoven leads I, II, and III. The sampling rate of the ECG is 500 Hz, the amplitude resolution is 24bit, and the bandwidth is 0–130 Hz. Both PPG and ECG, were recorded in the device's 8GB local flash memory using a secure GDF (General Data Format) (14).

**Figure 2:**
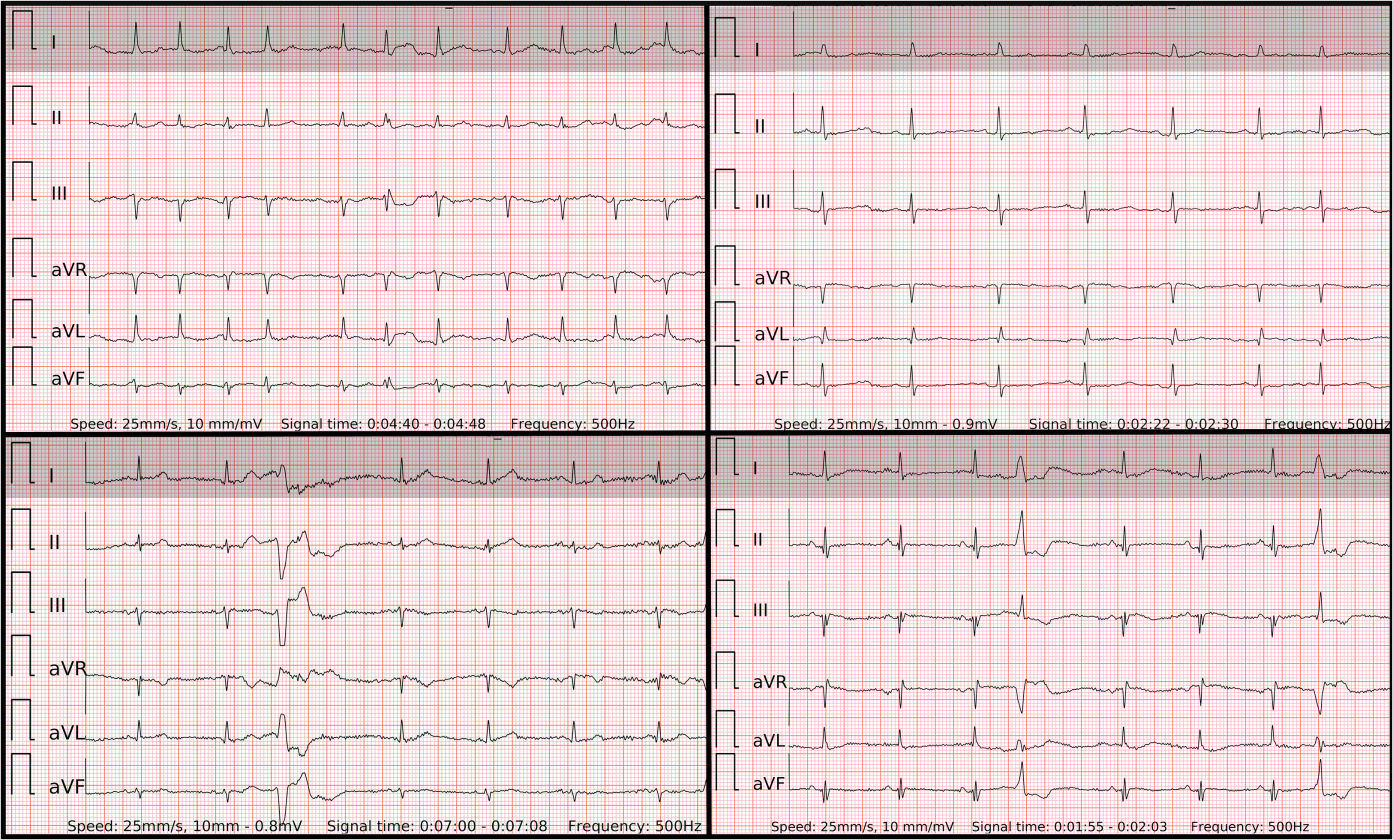
The 6-lead ECGs recorded by the wearable device with the examples of atrial fibrillation (top left panel); SR with frequent premature atrial contractions (top right panel); SR with frequent premature ventricular contractions with superior axis (lower left panel); SR with frequent premature ventricular contractions with inferior axis (lower right panel).

In order to display equivalent episodes of arrhythmia in a single-lead ECG, the six-lead wearable-recorded ECG was trimmed to a width of lead-I-like ECG (Figure 2). Thus, the accuracy of both methods was not influenced by any potential difference in the complexity of recording as it was exactly the same episode of arrhythmia. Reference ECG, single-lead ECG, and six-lead ECG from each patient were evaluated by two independent diagnosis-blinded cardiologists as “AF”, “SR”, or “Cannot be concluded”. In case of disagreement, a third cardiologist was asked to evaluate the case to make the final diagnosis.

### Data analysis

Continuous variables were reported as mean with standard deviation or median with interquartile range. Categorical variables were presented as counts and percentages. Detection performance was evaluated using sensitivity, specificity and accuracy. Due to the great dependence on the prevalence of disease, positive or negative predictive value were not evaluated. An independent sample Student's T-test or Mann-Whitney U test was applied to quantitative data. When the expected values in any of the cells of a contingency table were ≥5, a Chi-square test was applied for categorical data. Otherwise, a two-tailed Fisher's exact test was selected. Cramer's *V* was used to measure the association between the results of investigated diagnostic method and reference. Cohen's kappa was used to measure inter-rater agreement. Data was processed using the statistical package for the social sciences (27.0, SPSS Inc., Chicago, IL, USA).

## Results

**Figure 3:**
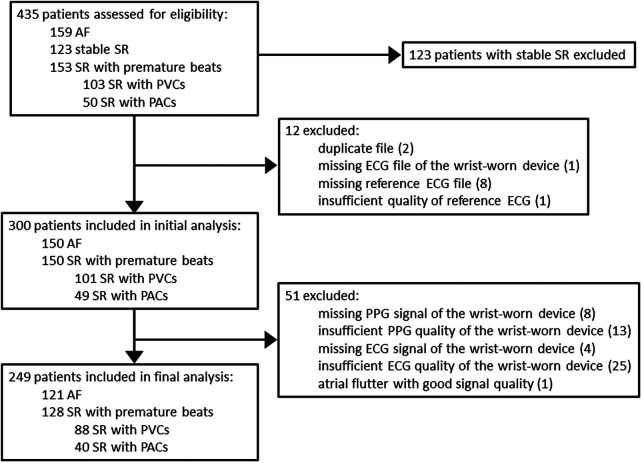
Flow chart of patients. AF, atrial fibrillation; SR, sinus rhythm; PVC, premature ventricular contraction; PAC, premature atrial contraction; ECG, electrocardiography; PPG, photoplethysmography.

In this substudy of the DoubleCheckAF trial, the initial assessment group for eligibility constituted 435 patients (Figure 3), of which 123 patients with stable SR were excluded. In addition, 12 recordings with duplicates or other similar issues of data logistics were excluded. Among the rest of the recordings, 1.3% (4/300) were with missing ECG signal of the prototype wrist-worn device and 8.3% (25/300) were with insufficient ECG quality of the prototype wrist-worn device. The final analysis included 249 patients, i.e., 121 patients with AF and 128 patients in the control group of SR with frequent premature beats, which consisted of dominant PVCs (*n* = 88) or PACs (*n* = 40).

In the control subgroup of SR with PACs and PVCs, the burden of premature beats per minute constituted a total of 5.5 beats/min (3, 13.9) and 6.7 beats/min (2.7, 16.4), respectively (Table 1). Patients with frequent PVCs were more likely to present with bigeminy/trigeminy (31.8%, 28/88) and less likely with runs of ≥3 beats (5.7%, 5/88) compared to patients with frequent PACs (7.5%, 3/40, and 17.5%, 7/40, respectively). These parameters represent not only just discrete single premature beats in both control subgroups but also the grouped extrasystoles or very frequent bigeminy/trigeminy episodes, which cause high irregularity.

**Table 1:** Baseline characteristics.

Characteristic	AF (*n *= 121)	SR with frequent premature contractions (*n *= 128)	
		SR with frequent PACs (*n *= 40)	SR with frequent PVCs (*n *= 88)
Age (years), mean ± SD	65.6 ± 11.2	70.9 ± 11.6	65.7 ± 15.0
Male, *n* (%)	64 (52.9)	20 (50)	49 (55.7)
Paroxysmal: persistent: permanent AF	101:14:6	NA	NA
**Type and frequency of premature contractions**			
Cases with frequent runs of ≥3 PACs/PVCs, *n* (%)	0 (0)	7 (17.5)	5 (5.7)
Cases with frequent bigeminy/trigeminy episodes, *n* (%)	0 (0)	3 (7.5)	28 (31.8)
PACs, median beats/min (IQR)	< 0.5	5.4 (2.6, 12.8)	<0.5
PVCs, median beats/min (IQR)	<0.5	<0.5	5.6 (2.4, 16.4)
Total, median beats/min (IQR)	<0.5	5.5 (3, 13.9)	6.7 (2.7, 16.4)
**CHADS_2_VASc risk score (categorical)**			
0–1, *n* (%)	37 (30.6)	1 (7.1)^a^	0 (0)^b^
2–4, *n* (%)	64 (52.9)	8 (57.1)^a^	13 (76.5)^b^
≥5, *n* (%)	20 (16.5)	5 (35.7)^a^	4 (23.5)^b^
CHADS_2_VASc risk score (quantitative), mean ± SD	2.7 ± 1.7	4 ± 2.1^a^	3.6 ± 1.2^b^
HAS-BLED score, mean ± SD	0.9 ± 0.8	1 ± 0.7^a^	1.7 ± 1.2^b^
OAC, *n* (%)	91 (75.2)	10 (25)	13 (14.8)
DOAC, *n* (%)	67 (55.4)	6 (15)	9 (10.2)
Warfarin, *n* (%)	23 (19)	4 (10)	4 (4.5)
LMWH, *n* (%)	1 (0.8)	0 (0)	0 (0)

AF, atrial fibrillation; SR, sinus rhythm; PVC, premature ventricular contraction; PAC, premature atrial contraction; OAC, oral anticoagulant; DOAC, direct oral anticoagulant; LMWH, low molecular weight heparin; IQR, interquartile range.

^a^ Calculated for patients with a history of AF, thus the denominator is 14.

^b^ Calculated for patients with a history of AF, thus the denominator is 17.

In the group of AF the median duration of an ECG and the median number of six-lead or single-lead ECG recordings per patient was 166.5 s (130, 222.5) and 1 recording (1, 1), respectively.

In the control group of frequent PACs/PVCs the median duration of an ECG and the median number of six-lead or single-lead ECG recordings per patient was 156 s (125.5, 209.8) and 1 recording (1, 2), respectively.

Accordingly, the duration of PPG signal per patient was 1,358 seconds (892, 2,206) in patients with AF and 1,113 seconds (915.8, 1,718.8) in patients with frequent premature beats.

### Single-lead ECG, six-lead ECG and PPG-based algorithm for AF detection when controlled by SR with PACs and PVCs

When compared to the control group, AF detection based on six-lead ECG, single-lead ECG, and PPG-based detector yielded a sensitivity of 99.2% (95% CI: 95.4–100), 95.7% (95% CI: 90.3–98.6), and 94.2% (95% CI: 88.4–97.6), respectively (Table 2). Due to type I error, the specificity of the same diagnostic tools was 98.4% (95% CI: 94.4–99.8), 92.5% (95% CI: 86.2–96.5) and 94.5% (95% CI: 89.1–97.8), respectively. The six-lead ECG demonstrated the highest overall accuracy with 98.4% (95% CI: 89.1–97.8), followed by the PPG-based detector with 94.5% (95% CI: 90.9–97) and single-lead ECG with 92.5% (95% CI: 88.4–95.5).

**Table 2:** Diagnostic measures of the wrist-worn device for AF detection controlled by SR with PVCs/PACs.

Measure	Single-lead ECG	Six-lead ECG	PPG-based detector
Sensitivity^a^, % (95% CI)	95.7 (90.3–98.6)	**99.2** (95.4–100)	94.2 (88.4–97.6)
Specificity^a^, % (95% CI)	92.5 (86.2–96.5)	**98.4** (94.4–99.8)	94.5 (89.1–97.8)
Accuracy^a^, % (95% CI)	92.5 (88.4–95.5)	**98.4** (96.0–99.6)	94.5 (90.9–97)
False positive cases, *n* (%)	**9/120 (7.5)**	2/127 (1.6)	7/128 (5.5)
False negative cases, *n* (%)	5/117 (4.3)	1/119 (0.8)	**7/121 (5.8)**
Cannot be concluded by a physician, *n* (%)	**12/249 (4.8)**	3/249 (1.2)	NA
Cramer's *V*, PACs subgroup	0.803, *P* < 0.001	**0.950**, *P* < 0.001	0.823, *P* < 0.001
Inter-rater agreement, PACs subgroup^b^	0.803, *P* < 0.001	**0.950**, *P* < 0.001	NA
Cramer's *V*, PVCs subgroup	0.918, *P* < 0.001	**0.990**, *P* < 0.001	0.903, *P* < 0.001
Inter-rater agreement, PVCs subgroup^b^	0.918, *P* < 0.001	**0.990**, *P* < 0.001	NA
AUC, PACs subgroup (95% CI)	0.898 (0.849–0.946)	**0.971** (0.948–0.994)	0.921 (0.881–0.962)
AUC, PVCs subgroup (95% CI)	0.961 (0.935–0.987)	**0.996** (0.988–1.00)	0.954 (0.926–0.982)
PACs/PVCs in false positive cases, median beats/min (IQR)	18.8 (11.6, 22.6)	**64.3** (41.2, 87.4)	13.2 (10, 41.2)

AF, atrial fibrillation; SR, sinus rhythm; PVC, premature ventricular contraction; PAC, premature atrial contraction; IQR, interquartile range. Both wearable-recorded ECGs were interpreted manually by diagnosis-blinded cardiologists. The PPG-based AF detector operated automatically.

^a^ Calculated for the overall control group of SR with PACs and PVCs.

^b^ Measured as Cohen's kappa.

The highest values are in bold.

False positive cases were more common for single-lead ECG (9/120, 7.5%) or tended to be more common for PPG-based detector (7/128, 5.5%) compared to six-lead ECG (2/127, 1.6%) (*P *= 0.02 and *P *= 0.08, respectively).

**Figure 4:**
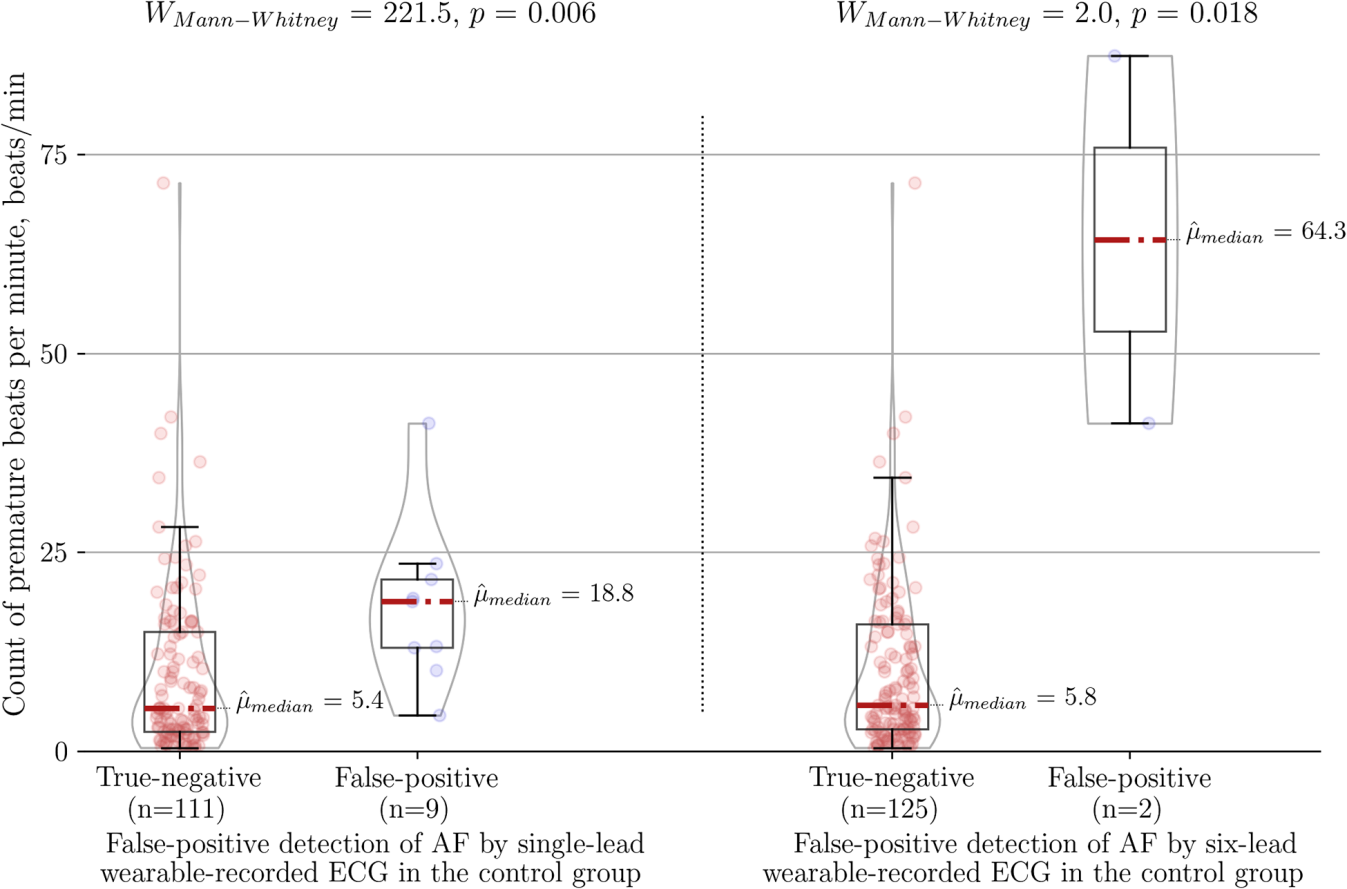
Association between count of premature beats per minute and type I error of the wearable-recorded single-lead ECG (left panel) vs. six-lead ECG (right panel) in the control group of SR with frequent premature beats. AF, atrial fibrillation.

The higher number of premature beats per minute was the main factor associated with false positive cases in comparison to true negative cases for each diagnostic method, namely the single-lead ECG (18.80 vs. 5.40 beats/min, *P* < 0.01), the six-lead ECG (64.3 vs. 5.8 beats/min, *P* = 0.018) and the PPG-based detector (13.20 vs. 5.60 beats/min, *P* = 0.05) (Figure 4). Of note, six-lead ECG was the most robust tool as it required 3.4 times more premature beats to result in a false positive outcome compared to single-lead ECG and 4.9 times more premature beats compared to the PPG-based detector. A single-lead ECG (12/249) was more frequently labeled “Cannot be concluded” than six-lead ECG (3/249) (*P* = 0.01).

There was no trend of AF with higher rates of beats per minute in false negative cases. The median beats per minute in false negative cases of PPG-based detector (7/121) was 92 bpm (58, 116). Accordingly, in a single false negative case of six-lead ECG (1/119) the median was 76 bpm and in 5 cases of single-lead ECG the median was 92 bpm (92, 94).

### Single-lead ECG, six-lead ECG, and PPG-based algorithm for AF detection when controlled by SR with frequent PACs

**Figure 5:**
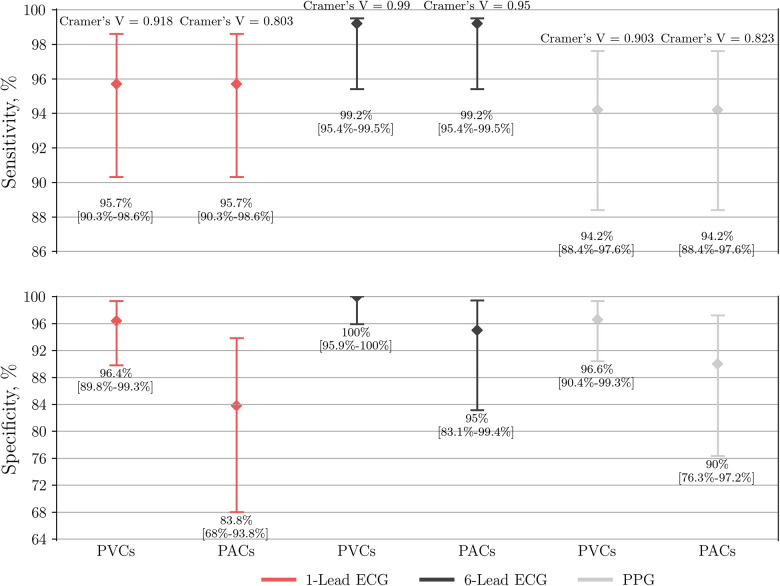
Performance of single-lead ECG (*n* = 237), six-lead ECG (*n* = 246) and the PPG-based algorithm (*n* = 249) to detect AF. The group of AF is compared to either a control subgroup of SR with frequent PVCs or PACs. PPG, photoplethysmography; ECG, electrocardiography; AF, atrial fibrillation; SR, sinus rhythm; PVC, premature ventricular contraction; PAC, premature atrial contraction.

When compared to the control subgroup of PACs, the specificity of AF detection by six-lead ECG, single-lead ECG, and PPG-based detector dropped to 95% (95% CI: 83.1–99.4), 83.8% (95% CI: 68–93.8), and 90% (95% CI: 76.3–97.2), respectively (Figure 5). Interestingly, further analysis of single-lead ECGs (AUC 0.898; Cramer's *V* association 0.803, *P* < 0.001; inter-rater agreement Cohen's kappa 0.803, *P* < 0.001) showed lower diagnostic value not only compared to six-lead ECG (AUC 0.971; Cramer's *V* association 0.950, *P* < 0.001; inter-rater agreement Cohen's kappa 0.950, *P* < 0.001), but also lower than PPG-based detection (AUC 0.921; Cramer's *V* association 0.823, *P* < 0.001).

### Single-lead ECG, six-lead ECG, and PPG-based algorithm for AF detection when controlled by SR with frequent PVCs

When compared to the control subgroup of PVCs, the specificity of AF detection by six-lead ECG, single-lead ECG, and PPG-based detector yielded a specificity of 100% (95% CI: 95.9–100), 96.4% (95% CI: 89.8–99.3), and 96.6% (95% CI: 90.4–99.3), respectively (Figure 5). In this case the diagnostic value of single-lead ECG (AUC 0.961; Cramer's *V* association 0.918, *P* < 0.001; inter-rater agreement Cohen's kappa 0.918, *P* < 0.001) was lower compared to six-lead ECG (AUC 0.996; Cramer's *V* association 0.990, *P* < 0.001; inter-rater agreement Cohen's kappa 0.990, *P* < 0.001), but non-inferior to PPG-based detector (AUC 0.954; Cramer's *V* association 0.903, *P* < 0.001).

## Discussion

### Major findings

This study investigates the diagnostic accuracy of the first wrist-worn device with a PPG-based AF detector and intermittent simultaneous six-lead standard-limb-like ECG for manual rhythm confirmation by a physician. The main focus of the current analysis is a head-to-head comparison of single-lead and six-lead ECGs as well as the automatic PPG-based AF detector of the same wearable device. Major findings are: (1) comparing to any control subgroup of SR with premature beats (PACs or PVCs) the diagnostic value of six-lead ECG was significantly superior to single-lead ECG and PPG-based AF detector both regarding type I and type II errors. (2) The sensitivity of single-lead ECG was slightly higher compared to PPG-based detector in both control subgroups. (3) Single-lead ECG demonstrated lower specificity not only vs. six-lead ECG but also vs. PPG-based automatic AF detection when controlled by a subgroup of frequent PACs. (4) The specificity of single-lead ECG and PPG-based detector were equivalent when controlled by a subgroup of frequent PVCs. (5) The number of premature beats per minute was the main factor associated with false positive cases compared to true negative cases for all diagnostic tools. (6) Six-lead ECG was the most robust tool as it required 3.4 times more premature beats to result in a false positive outcome compared to single-lead ECG and 4.9 times more premature beats compared to the PPG-based detector. (7) Based on previous findings, the widespread use of single-lead ECGs recorded by smartwatches significantly increases the risk of type I error in populations with frequent premature contractions.

It is important to emphasize the choice of the control group in this study, which included SR with frequent premature contractions. Stable SR was excluded from the control group, which is in contrast to the vast majority of other mHealth studies (15–18). This choice was based on the DoubleCheck-AF trial, in which it was demonstrated that stable SR as an isolated control subgroup does not sufficiently challenge the specificity of diagnostic tools (11).

### Why six is smarter than one: impact of electrode contact in wearables and relation to the topographic anatomy of sinus node

The concept of an original Einthoven's triangle, generated by the contact of three electrodes and described by prof. W. Einthoven, explains why certain ECG leads of modern mHealth technologies maintain or decline the signal quality (19). In case of recording a single-lead ECG (i.e., lead-I-like in smartwatches), one insufficient contact on the left or right arm causes absence of ECG or artifacts which complicate the interpretation of ECG. In case of recording a six-lead ECG with three electrodes, one insufficient contact results in artifacts of two involved leads while leaving the third lead unaffected. This is the main practical reason why a wearable-recorded six-lead ECG outperformed the single-lead ECG to accurately differentiate AF and SR with frequent premature beats.

Another reason of the better performance of six-lead ECG vs. single-lead ECG relates to the location of the sinus node in the right atrium (RA). Chen X. et al. (20) performed the 3D electroanatomical mapping and investigated the earliest atrial activation (EAA), which represent the exit site of sinus node, in a population of patients with AF who were scheduled for superior vena cava (SVC) isolation. The EAA in a majority of patients with AF was located above the RA SVC junction 72/136 (52.9%), especially in a subgroup of persistent AF with a proportion of 26/43 (60.5%). Of those with EAA below RA SVC (64/136 (47.1%), the high position of EAA in RA was predominant and constituted 60/64 (93.8%). As a consequence, the high location of sinus node exit in individuals with AF or SR transfers to relevant wearable-recorded ECG features. The axis of *P* wave in SR is predominantly inferior and slightly less leftward. Accordingly, one of the main standard-limb-lead ECG features of SR is that the *P* wave amplitude in lead II comes out bigger than in lead I. Therefore, the usual *P* wave in lead-I-like ECG of smartwatches is not as apparent as in lead-II-like ECG. Suppose we put this small but relevant difference in *P* wave amplitude together with artifacts, which are quite common for all wearable-recorded ECGs. In that case, it partly explains why single-lead ECG was inferior to six-lead ECG to detect AF in the current study. In addition, even if a smartwatch is used to record a single-lead-II-like ECG (21, 22), it would arguably still be unable to outperform the six-lead ECG. Any single-lead ECG inevitably lacks the possibility to simultaneously check the reproducibility of suspected *P* waves throughout each of the six leads and exclude the mimicking artifacts.

These hypotheses are partly supported by another study of 220 patients (15), where manual interpretation of lead-II-like ECG by either Withings or Apple Watch (correct classification 54%) was numerically superior to the manual interpretation of lead-I-like ECG by Withings (28%, *P* = 0.076) or Apple Watch (33%, *P* = 0.246) for detection of atrial flutter. In addition, the six-lead ECG of Kardia 6l was the most accurate method for a correct diagnosis of atrial flutter in 63% of all cases (*P* < 0.001 compared to Withings and Apple Watch). Of note, no control group of SR patients with frequent PACs/PVCs was included.

### ECG examples of false negative, false positive and inconclusive cases in single-lead vs. six-lead ECGs

When the ECG signal has no major artifacts (Figure 2) presumably even one beat of PQRST complexes in any single-lead ECG could be sufficient to differentiate AF from SR with premature beats. However, the decisive real-world difference in diagnostic accuracy lies in ECGs with lower signal quality. In fact, artifacts are common not only in wearable-recorded ECGs but also in conventional ambulatory ECG monitoring. El-Sherif et al. (9) reported artifacts in 4.8% (48/1,000) and misinterpretations in 3.5% (35/1,000) of recordings in ambulatory ECG monitoring or telemetry. Of them, most artifacts were misclassified as pseudo-ventricular tachycardia or pseudo-AF/atrial flutter due to movement-generated repetitive waves, which hide real QRS or *P* waves. In addition, most misinterpretations were pseudo-ventricular tachycardia due to high rates of SVT/AF with bundle branch block.

**Figure 6:**
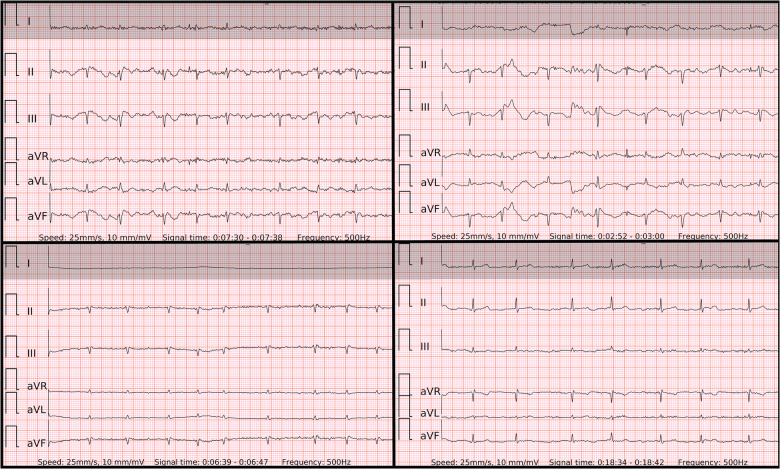
Problematic recordings of AF. False if interpreted by single lead-I-like ECGs (marked in gray) vs. correct if interpreted by six-lead ECGs. Top left panel: false negative due to artifacts mimicking P’ of runs of PACs in single-lead ECG, true positive in six-lead ECG with no reproducible *P* waves; Top right panel: cannot be concluded due to low amplitudes in single-lead ECG, true positive in six-lead ECG; Lower left panel: cannot be concluded due to isoelectric signal in single-lead ECG, true positive in six-lead ECG with no reproducible *P* waves; Lower right panel: false negative due to artifacts mimicking P’ of PACs and pseudo *regularly*-irregular R-R intervals in single-lead ECG, true positive in six-lead ECG with no reproducible *P* waves.

In our study, the sensitivity of six-lead ECG was superior to single-lead ECG. The lower ECG signal quality was predominantly present in isolated leads, such as lead-I-like. Occasionally the ECG recordings presented with repetitive artifacts in the usual location of *P* waves, also, R-R intervals were rather regularly-irregular, mimicking SR with PACs (Figure 6) in single-lead ECGs. These factors typically led to false negative outcomes in patients with AF in single-lead ECG and true positive detection in six-lead ECG. Interestingly, there was no trend of AF with higher rates of beats per minute in false negative cases as the median of beats per minute did not reach 100 bpm.

**Figure 7:**
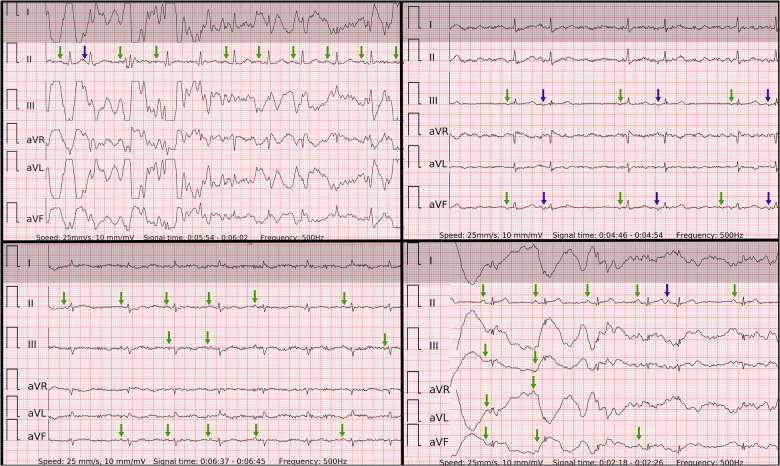
Problematic recordings of SR with PACs. False if interpreted by single lead-I-like ECGs (marked in gray) vs. correct if interpreted by six-lead ECGs. Top left panel: cannot be concluded due to artifacts in single-lead ECG, true negative in six-lead ECG with reproducible *P* waves of SR (green arrows) and *P*′ of PAC on the T wave (blue arrow); Top right panel: false positive due to artifacts masking small *P* waves and mimicking f waves in single-lead ECG, true negative in six-lead ECG with reproducible *P* waves of SR (green arrows) and P’ of PACs (blue arrows); Lower left panel: false positive due to artifacts masking small *P* waves and mimicking f waves in single-lead ECG, true negative in six-lead ECG with reproducible *P* waves of SR (green arrows); Lower right panel: cannot be concluded due to artifacts in single-lead ECG, true negative in six-lead ECG with reproducible *P* waves of SR (green arrows) and P’ of PAC after the T wave (blue arrow);.

**Figure 8:**
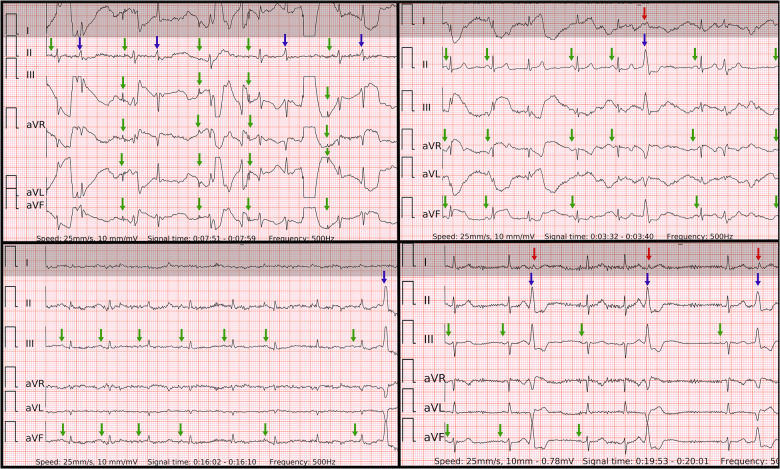
Problematic recordings of SR with PVCs. False if interpreted by single lead-I-like ECGs (marked in gray) vs. correct if interpreted by six-lead ECGs. Top left panel: cannot be concluded due to artifacts in single-lead ECG, true negative in six-lead ECG with reproducible *P* waves of SR with some artifacts (green arrows) and QRS of PVC or aberrancy (blue arrow); Top right panel: false positive due to artifacts masking small *P* waves and mimicking f waves as well as pseudo irregularly-irregular R-R intervals due to barely visible QRS of PVC (red arrow) in single-lead ECG, true negative in six-lead ECG with reproducible *P* waves of SR (green arrows) and big QRS of PVC with inferior axis (blue arrow); Lower left panel: cannot be concluded due to artifacts and small amplitudes in single-lead ECG, true negative in six-lead ECG with reproducible *P* waves of SR (green arrows) and QRS of PVC with inferior axis (blue arrow); Lower right panel: cannot be concluded due to artifacts and unclear irregularity type of R–R intervals due small QRS of PVCs (red arrows) in single-lead ECG, true negative in six-lead ECG with reproducible *P* waves of SR (green arrows), regularly-irregular R–R intervals and QRS of bigeminy/trigeminy PVCs with inferior axis (blue arrows).

**Figure 9:**
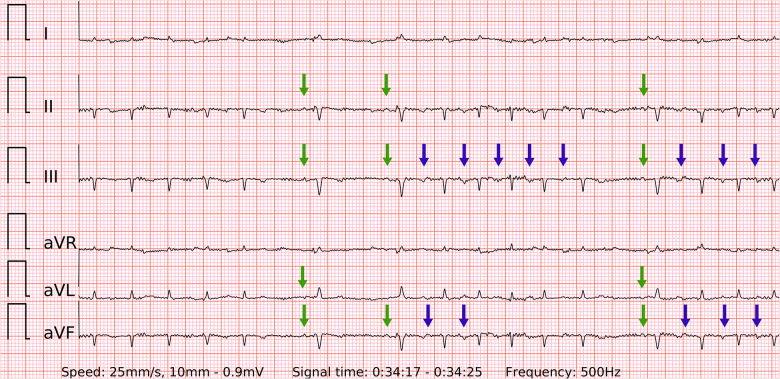
A problematic recording of SR with runs of PACs. A rare example of false positive in both single-lead and six-lead ECGs. Presumably due to the small amplitude of abnormal P’ waves (blue arrows) and lightly irregular R-R intervals during fast bursts of runs of PACs. The *P* waves of SR are visible and reproducible (green arrows), but overwhelmed by the previous findings.

The specificity of six-lead ECG was superior to single-lead ECG due to similar reasons. Firstly, likely poor contact on one of the electrodes led to a distorted ECG signal on two leads (one of which was usually lead-I-like ECG) of Einthoven's triangle. However, the remaining third lead stayed unaffected. Secondly, the amplitude of the *P* wave in lead-I-like appeared smaller compared to the *P* wave in lead-II-like ECG. Therefore, the six-lead ECG allowed to avoid false positive outcomes as opposed to single-lead ECG in both control subgroups of SR with frequent PACs (Figure 7) and PVCs (Figure 8). Few cases (2/127) with runs of PACs resulted in false positive outcome in both six-lead and single-lead ECGs, presumably due to the small amplitude of abnormal *P* waves and irregular R-R intervals during fast bursts of runs of PACs (Figure 9).

These ECG examples illustrate why of all three diagnostic tools the six-lead ECG was the most refractory to frequent premature contractions as well as the least likely to be labeled “Cannot be concluded”. As a future prospect, six-lead ECG has an additional advantage of potentially reconstructing the axis of both QRS complex and *P* wave. Although this is out of the scope of current study and no precordial leads are displayed, it could help identify the approximate location of arrhythmias with rare clinical presentation, such as existing ECG algorithms for idiopathic ventricular tachycardia/PVC or atrial tachycardia (23, 24).

### Results of other wearable devices with six-lead ECG for AF detection

To the best of our knowledge, there are two wearable devices capable of recording six-lead ECG with no wires: Kardia Mobile 6l (KM) and Istel HR 2000 (IS). Both of them essentially work as event recorders, which provide intermittent six-lead ECG for opportunistic screening of AF. In contrast to the wrist-worn device used in this study, they have no PPG-based AF detector for continuous screening of AF (12).

Krzowski et al. (16) analyzed 98 patients with a head-to-head comparison of KM and IS after manual interpretation by physicians. For diagnosing SR, KM yielded a sensitivity of 88.1% and a specificity of 89.7%. IS yielded 91.5% and 84.6% sensitivity and specificity, respectively. The sensitivity of KM in detecting AF was higher than IS (86.4% vs. 77.3%), but their specificity was comparable (97.4% vs. 98.7%). Notably, the control group in this study included patients with only SR and no premature contractions.

Scholten et al. (15) presented reproducible results in line with our findings. The manual interpretation of KM six-lead ECG was superior (sensitivity 98.9%, specificity 96.7%) to manual interpretations of single-lead ECG of Withings (sensitivity 95.4%, specificity 94.9%) and Apple Watch (sensitivity 96.2%, specificity 94.4%) for AF detection. Importantly, there was no dedicated control group of SR with premature beats, only patients with stable SR after electrical cardioversion were included in the control group.

These studies produce comparable results, which support the idea of six-lead ECG diagnostic superiority to single-lead ECG for AF detection. Nevertheless, the above-mentioned studies were not designed to include a dedicated control group of patients in SR with frequent premature contractions.

### Limitations

Several limitations apply to the study. Firstly, it is a substudy of DoubleCheckAF, which originally was not intended for recording a single-lead ECG. In order to display a single-lead ECG, the six-lead wearable-recorded ECG was trimmed to a width of lead-I-like ECG. However, there is also an advantage to it as the accuracy of both diagnostic tools was not influenced by any potential difference in the complexity of recording since it was exactly the same episode of arrhythmia. Secondly, as outlined in Figure 3 some patients were excluded due to issues with data logistics, insufficient signal quality and other reasons. This could cause additional costs or visits for patients in real-life conditions. Thirdly, since the participants in the presented study were White the performance of PPG detector could not be generalized for other skin pigmentations. Finally, all recordings were done in a hospital after a short explanation by a physician. As highlighted by the EHRA practical guide the implementation of wearables requires improved digital health literacy among patients and healthcare personnel (8). In an outpatient setting the users have to move up the learning curve, and hence the real-world accuracy may differ, particularly when starting to use a new device.

## Conclusions

A six-lead ECG recorded by a wearable with no wires demonstrated the superior diagnostic value of AF detection compared to a single-lead ECG and automatic PPG-based AF detection when controlled by patients with any type of frequent premature contractions. The performance of a single-lead ECG was inferior to a PPG-based AF detector when controlled by patients with frequent PACs and non-inferior when controlled by patients with frequent PVCs. The risk of type I error due to the widespread use of single-lead ECGs of smartwatches in populations with frequent premature beats is significant.

## Data Availability

The raw data supporting the conclusions of this article will be made available by the authors, without undue reservation.
